# Parents’ awareness and perceptions of the Change4Life 100 cal snack campaign, and perceived impact on snack consumption by children under 11 years

**DOI:** 10.1186/s12889-022-12789-7

**Published:** 2022-05-19

**Authors:** Rhiannon E. Day, Gemma Bridge, Kate Austin, Hannah Ensaff, Meaghan S. Christian

**Affiliations:** 1 School of Clinical and Applied Sciences, Leeds Beckett University, CL615A, City Campus, Leeds, LS1 3HE UK; 2 Leeds, UK; 3 Nutritional Sciences and Epidemiology, School of Food Science and Nutrition, University of Leeds, Leeds, LS2 9JT UK; 4 Department of Nutrition, Dietetics & Food, Monash University, Be Active Sleep Eat (BASE) Facility, Level 1, 264 Ferntree Gully Road, Notting Hill, VIC 3168 Australia

**Keywords:** Childhood, Intervention, Nutrition, Obesity, Snacking, Public health

## Abstract

**Background:**

Childhood obesity is a pertinent public health problem in the UK. Consumption of free sugars has been associated with the development of obesity. In 2018, the Change 4Life (C4L) 100 cal snack campaign was launched with the slogan ‘100 calorie snacks, two a day max’, aiming to encourage parents to choose lower sugar, fat and calorie snacks for their children. This study aimed to examine how the campaign has been perceived by parents.

**Methods:**

An online survey was developed to explore parent awareness, perceptions and understanding of the C4L 100 cal snack campaign. Respondents were recruited via Leeds City Council, posters displayed at primary schools and children’s centres across Leeds and via social media. Paper surveys were also shared with voluntarily led playgroups. Survey data was analysed using descriptive statistics. Thematic analysis was performed on open text responses.

**Results:**

Three hundred forty-two 342 respondents completed the survey. Just over half of the respondents had come across the campaign, most seeing the leaflet or a television advert. Over two-thirds of respondents ‘agreed’ or ‘strongly agreed’ that the campaign caught their attention. A similar proportion ‘agreed’ or ‘strongly agreed’ that the campaign informed them about 100 cal snacks and just over a half thought it was memorable. Most respondents used positive language to describe the campaign, but there was no clear consensus of a perceived positive impact on healthier snack purchasing, nor preparing more 100 cal snacks at home. Respondents provided examples of how the campaign could be improved to positively impact eating behaviours: better publicity and information delivery; healthier snack examples made more visible; improved nutritional labelling and access to healthier products in supermarkets (availability, promotion, display, choice).

**Conclusions:**

The C4L 100 cal snack campaign was perceived positively by parents and carers, with many agreeing that the campaign was informative and memorable. However, there was no agreement in terms of the parents reporting an impact of the campaign on behaviour change and healthier snack habits. Future social marketing campaigns could be improved through more formal pilot testing to assess the understanding and acceptance of the campaign amongst the target audience.

**Supplementary Information:**

The online version contains supplementary material available at 10.1186/s12889-022-12789-7.

## Background

Childhood obesity is a pertinent public health challenge both globally [1] and in the United Kingdom (UK) [2]. There is concern about the increasing prevalence of childhood obesity [3], as it tracks into adolescence and adulthood [4, 5], and can lead to adverse health outcomes, such as high blood pressure and type 2 diabetes [6–8]. The latest data from the National Child Measurement Programme (NCMP) in England in 2019/20 [9], indicates that in Reception class (aged 4–5 years), almost a quarter of children (23%) are living with overweight or obesity, with an increase of obesity prevalence to 10%. By the end of primary school (age 10–11 years), over a third of children (35%) are living with overweight or obesity, with obesity prevalence increasing to 21%. With childhood obesity prevalence increasing, the need for action to identify targets for prevention and treatment remains high [10].

It is understood that the causes of obesity are multifactorial and complex [11], but diet is a particularly well-established modifiable risk factor [12]. Excessive consumption of calories, and in particular free sugars [13, 14], has been associated with the development of obesity [15]. Moreover, research indicates that overconsumption of calories is one of the most significant contributing factors in becoming overweight, with many adults in the UK consuming 200–300 extra calories a day above recommended daily guidelines, whilst children living with overweight or obesity are consuming up to 500 more calories than recommended each day [16]. Many of these excess calories can come from snacking occasions throughout the day. Many snack foods consumed by children of all ages are highly processed, energy-dense, high in sugar and of low nutritional quality [17, 18]. Though data on snacking and obesity in children are limited and equivocal, there is evidence that children who snack on such products frequently, consume greater energy [19], have poorer quality diets, and exhibit other risk factors for excessive weight gain [18]. Furthermore, a secondary analysis of data from the UK National Diet and Nutrition Survey (NDNS) (Years 5 and 6 combined) by Public Health England (PHE) [20], indicates that children (aged 4–18 years) are getting half their sugar intake (51.2%), currently around 7 sugar cubes (approximately 21 g) a day, from energy-dense snack foods (such as biscuits and cakes) and sweetened soft drinks, leading to obesity and dental decay. Moreover, children were consuming at least 3 energy dense, sugary snacks and sugary drinks a day, with around a third consuming 4 or more, resulting in consumption of around three times more sugar than is recommended [20]. Given that snacking habits are established during childhood and often persist into adulthood [21], snacking on foods and drinks of low nutritional quality should be discouraged at an early age. Moreover, research has shown that targeting snack occasions may be specifically beneficial in children [10].

Action is required to improve dietary intake, with childhood an important opportunity to improve long term intake and reduce the long-term risk of obesity and other non-communicable diseases (NCDs) [22]. Such action needs upstream approaches such as reformulation, and downstream approaches that aim to inform the public, change opinion and build support for change [23]. ‘Change4Life’ (C4L) is an example of a downstream social marketing campaign that was launched in 2009 by PHE, as part of the UK government’s strategy to reduce obesity [24]. The C4L campaign ran across television, print and poster advertising, to encourage target groups to reduce calorie intake and develop healthier eating habits (reductions in foods high in added sugar and fat (HFHS), a more regular meal pattern, less snacking, and increased fruit and vegetable intake), be aware of the health risk of excess body fat, and participate in regular physical activity and reduce sedentary time [25]. In January 2018, an extension to the initial C4L campaign was launched; the ‘C4L 100 calorie snack campaign’ ran with the slogan ‘100 calorie snacks, two a day max’ [26]. A national advertisement campaign (written information, website and television advert) was delivered for 2 months. The webpage offered advice to parents around packaged snacks to look for “100 calories, two a day max” and to make quick decisions on packaged snacks, by providing recommended examples of snacks to prepare at home and while away from home. It also provided information on calories (including where to locate calories labelling), sugar content and basic instructions on how to use traffic light labelling. Alongside the campaign and website, a food scanner app was launched to show the calorie, salt, sugar and fat content of foods, with the aim of making healthier choices easier [20].

To the best of our knowledge, no previous work has explored the C4L 100 cal snack campaign, or how it has been perceived by parents. Previous research has evaluated the impact of other branches of the C4L campaign, such as ‘Sugar Smart’ [23] on dietary behaviours, and has indicated an increased awareness of the campaign, but little impact on attitudes or behaviour [27], or that improved behaviour such as sugar reduction could not be sustained [23]. It is important to evaluate social marketing campaigns to both inform the development of future public health focussed initiatives and to assess the value for money of existing campaigns due to their use of public funds [28]. As a result, the current study aimed to assess parent awareness, perceptions and understanding of the C4L 100 cal snack campaign, and how children’s eating behaviours may have changed as a result of adjusted food practices due to the campaign.

## Methods

### The survey

An online survey was developed to explore two elements: 1) parent perceptions of their child’s snacking and mealtime behaviours in and outside of the home, and 2) parent awareness, perceptions and understanding of the C4L 100 cal snack campaign launched in 2018 in the UK. The findings of element 1) are discussed elsewhere (Bridge G, Day R, Armstrong B, Christian M: Family meals with young children: a survey study of family mealtime characteristics among British families with children under 11 years old, unpublished). This paper describes the findings related to element 2), the C4L 100 cal campaign. The survey was developed and shared with parents or carers of children aged up to 11 years old, who were living in the UK and over 18 years of age. Respondents were asked to answer survey questions about their youngest child if they had more than one child.

The survey was constructed using Qualtrics software 2020 (Qualtrics, Provo, UT), an online platform that facilitates the collection and analysis of data. The survey is included as a supplementary file (Additional file 1). The survey was developed and piloted for completion online (only one survey to be completed per family), with an appropriate format and layout incorporated into the design. The first part of the survey was designed by the research team, informed by response categories from a survey commissioned by PHE in 2018; ‘Public Perceptions and Awareness of Public Health England’s reduction Programmes’ [29]. The second part of the survey, the findings of which are discussed in this paper, explored four areas: 1) perceptions of the C4L 100 cal snack campaign relating to awareness of advertising, promotional materials and webpages relating to the C4L 100 cal snack campaign, 2) understanding of 100 cal snack campaign information; the impact of the campaign on child’s snack behaviours, 3) and recommendations for healthy snack information for parents. Only those who had seen the campaign, as assessed by responding yes to the question ‘have you seen the campaign?’ were able to answer this part of the survey. Respondents who said ‘no’ were redirected to the final block of questions in the survey. The final section obtained demographic, socioeconomic information and postcode data (so that the Index of Multiple Deprivation could be assigned). A paper-based version of the survey was piloted with a group of parents (*n* = 10) attending a community playgroup in Leeds and subsequently piloted online with a further sample of parents (*n* = 5). Minor changes were made to layout and wording for clarification before the survey was launched online.

The link to the online survey was advertised (via QR code on a poster) to primary schools across Leeds, via a contact at Leeds City Council. The link was also advertised via posters displayed at children’s centres across Leeds and on social media such as Netmums, Mumsnet, Facebook, Twitter, and on the Leeds National Childbirth Trust Facebook page. The survey was accessible from July 7th 2019 to October 24th 2019. To increase the diversity of the sample, paper surveys were also shared with three voluntary led playgroups in Leeds. Surveys were completed by carers or parents of a child aged up to 11 years, respondents were asked to think about their youngest child when completing the survey. To maximize participation and completion of the survey, most questions were not compulsory. Therefore, response numbers to each question vary.

### Data analysis

A summary report of findings was exported from Qualtrics (2020) into Microsoft Excel. The data was assessed using descriptive statistics such as counts, means and percentages. Percentages are presented to one decimal place or as whole numbers when *N* < 100 participants. Microsoft Excel (2020) was also used to create graphs and tables to explore the data. The open text responses from respondents were analysed by thematic analysis, informed by Braun and Clarke (2006) [30]. This process involved becoming familiar with the responses by reading and re-reading all responses. This was followed by coding all responses into themes, and then grouping these into meaningful categories. This was carried out by one researcher (RED), with agreement of themes by a second (GB). Due to the small volume of qualitative data, Microsoft Excel (2020) was used to manage the data during the analysis. Initial exploration of word frequencies was conducted using word clouds (with the largest words generated for those appearing most in the open text responses) and this provided an initial assessment of responses to open text items.

### Ethics

Ethical approval was provided by the Leeds Beckett University School of Clinical and Applied Sciences ethics review committee (reference number 54329). All methods were performed following the relevant guidelines and regulations. An information sheet at the start of the survey made respondents aware of how the data would be used. All respondents were given a participant information sheet and were asked to read and provide informed consent before answering any survey questions. Respondents were reminded that they were free to withdraw from the survey at any point up to data analysis. To encourage participation a free prize draw of a £50 high street shopping voucher was offered. Respondents wishing to be entered were asked to provide an email address.

## Results

### Respondent characteristics

The total number of respondents to the survey was 342. Table 1 presents the demographic and socioeconomic characteristics of the survey respondents. Not all respondents completed the demographic questions as they were kept optional in the survey. Most respondents were mothers (*n* = 288, 91.9%), with a mean age of 38 years (SD, 6.1, range 22–57 years). A large proportion of the sample had at least two children (*n* = 219, 70.1%). The mean age of the respondents’ youngest child was 5.1 years old (SD 3.0, range 0–11 years). The majority of respondents were born in the United Kingdom (*n* = 272, 90%), and around three-quarters of the sample were living in Leeds (*n* = 223, 77.2%). The majority were from White British backgrounds (*n* = 283, 93.7%); this is higher than the White-British population in Leeds (73.9%) [31] and the national average (86.0%) [32]. Over 70% of the sample had at least a level 4 qualification (degree, higher degree or professional qualification). This is much higher than the Leeds average (40.1%) and the national average (40.0%) [33]. Over a quarter of the sample were from the 20% most deprived areas (IMD quintile 1) in the UK (28%), similar to the average of 31% of the population for the Leeds area and 20% nationally [31].

**Table 1:** Characteristics of survey respondents

Demographic variables		
**Relationship to child (n %** ^a^ **)**		
Mother	283	91.9%
Father	20	6.5%
Grandparent	1	0.3%
Carer	2	0.6%
Other (stepmother)	2	0.6%
**Total**	**308**	100%
**Gender (n %)**		
Male	21	6.8%
Female	288	93.2%
**Total**	**309**	100%
**Number of children in the household (n %)**		
1	301	100%
2	211	70.1%
3	62	20.6%
4	31	10.3%
5	11	3.7%
**Highest education qualification (n %)**		
Less than 5 GCSEs or equivalent (e.g. O levels)	15	4.9%
5+ GCSEs (grades A^a^ - C) or equivalent (e.g. NVQ level 2)	23	7.5%
2+ A levels or equivalent (e.g. NVQ level 3)	44	14.4%
Degree (e.g. BSc)	85	27.9%
Higher degree or equivalent (e.g. PhD, PGCE)	79	25.9%
Professional qualifications (e.g. teaching, nursing)	51	16.7%
No qualifications	7	2.3%
Other (graduate higher diploma)	1	0.3%
**Total**	**305**	**100%**
**Country of birth (n %)**		
UK (England, Wales, Scotland or Northern Ireland)	272	90.0%
Other country	30	10.0%
**Total**	**302**	**100%**
**Region (n %)**		
Leeds	223	77.2%
Outside of Leeds	66	22.8%
**Total**	**289**	**100%**
**Ethnic background (n %)**		
White	283	93.7%
Mixed/Multiple ethnic background	6	2.0%
Asian/Asian British	7	2.3%
Black/African/Caribbean/Black British	3	1.0%
Other (Japanese, Vietnamese)	3	1.0%
**Total**	**302**	**100%**
**IMD** ^b^ **(n %)**		
1st	85	28.2%
2nd	59	19.6%
3rd	30	10.0%
4th	38	12.6%
5th	46	15.3%
Unknown/Unclassified	43	14.3%
**Total**	**301**	**100%**

^a^Percentages may not add up to 100% due to rounding

^b^IMD –deprivation quintiles score neighbourhoods from 1st (most deprived 20%) to 5th (least deprived 20%))

*Note*: Not all respondents provided completed the demographic questions as they were kept optional in the survey

### Awareness of Change4Life 100 cal snack campaign

Just over half of respondents who answered the question, stated that they had come across the C4L “100 calorie snacks, two a day max” campaign (54.7%, *n* = 187). Therefore, only these respondents were able to answer subsequent questions about the campaign, giving a smaller number of respondents to each question. When asked where they had seen or heard the phrase ‘look for 100 calorie snacks, two a day max’, 310 options were selected (respondents could choose as many as appropriate). Table 2 demonstrates that the most common responses were a C4L leaflet (*n* = 85, 27.4%) or a television advert (*n* = 61, 19.7%), followed by a social media advert (*n* = 48, 15.5%) and the C4Lwebsite (*n* = 45, 14.5%).

**Table 2:** Awareness of the 100 cal snack campaign

	N (%) of respondents^a^
**Where respondents reported seeing the campaign**	
C4L leaflet	85 (27.4%)
Television advert	61 (19.7%)
Social media advert	48 (15.5%)
C4L website	45 (14.5%)
Radio advert	24 (7.7%)
Supermarket	23 (7.4%)
Other (e.g. children’s centres and schools)	23 (7.4%)
**Total number of responses**	**310**
**Number of times they had seen the campaign**	
None	21 (11.5%)
Once	49 (26.8%)
2–3 times	76 (41.5%)
4–5 times	19 (10.4%)
6 or more times	18 (9.8%)
**Total number of responses**	**183**
**Where respondents reported seeing/receiving a leaflet about the campaign**	
Primary school	86 (62.8%)
GP surgery/ health centre	13 (9.5%)
Health professional	10 (7.3%)
Library	7 (5.1%)
Children’s centre	6 (4.4%)
Leisure centre	6 (4.4%)
Pharmacy	4 (2.9%)
Other (at work, through the post)	5 (3.7%)
**Total number of responses**	**137**

^a^Percentages do not always add up to 100 due to rounding

When asked how many times they saw the campaign in total (could only select one answer), 183 respondents provided an answer. Table 2 indicates that most saw it 2–3 times (*n* = 76, 41.5%). Some reported seeing the campaign 6 or more times (*n* = 36, 19.6%), whilst 9.8% (*n* = 18) reported that they had never seen the campaign. When asked if they had seen or received a leaflet about the campaign (could only select one answer), just over half of the 184 respondents to the question stated that they had seen a leaflet (*n* = 109, 59.2%). When asked where they had seen or received the leaflet (could select as many options as appropriate), the most common response was from primary school (*n* = 86, 62.8%), followed by GP surgery/health centre (*n* = 13, 9.5%) or health professional (*n* = 10, 7.3%), as indicated in Table 2.

### Perceptions of 100 kcal snack campaign

**Fig. 1:**
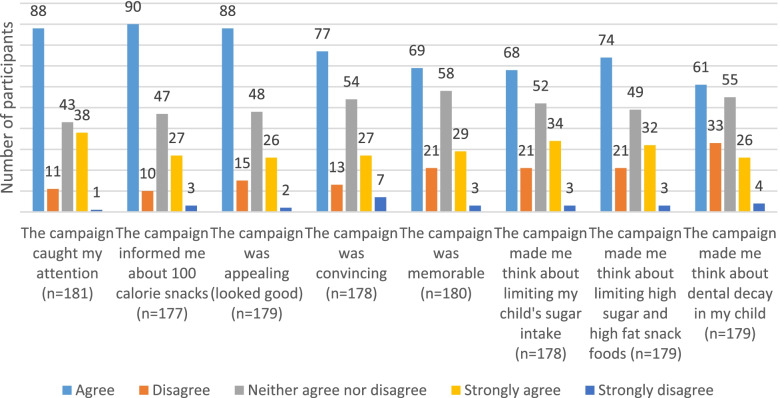
Respondents’ agreement with a series of statements about the campaign

The respondents were asked about their perceptions of the campaign through their agreement with a series of statements (summarised in Fig. 1). Number of respondents to each question varied as questions were optional and again, only those who had seen the campaign could answer. Over two-thirds of the 191 respondents to the question ‘agreed’ or ‘strongly agreed’ that the campaign caught their attention (*n* = 126, 69.6%). A similar proportion ‘agreed’ or ‘strongly agreed’ that the campaign informed them about 100 cal snacks (*n* = 117, 66.0%,), and just over a half thought it was memorable (*n* = 102, 54.4%). Of the 179 respondents who completed the following questions, just under a third ‘agreed’ or ‘disagreed’ that the campaign was appealing (looked good) (*n* = 114, 63.7%). A small majority ‘agreed’ or ‘strongly agreed’ that it was convincing (*n* = 104, 58.5%). Over half of the respondents ‘agreed’ or ‘strongly agreed’ that the campaign made them think about limiting high sugar and high fat snack foods for their child (*n* = 106, 59.2%), and just under a half of respondents ‘agreed’ or ‘strongly agreed’ that it made them think about dental decay in their child (*n* = 87, 48.6%).

**Fig. 2:**
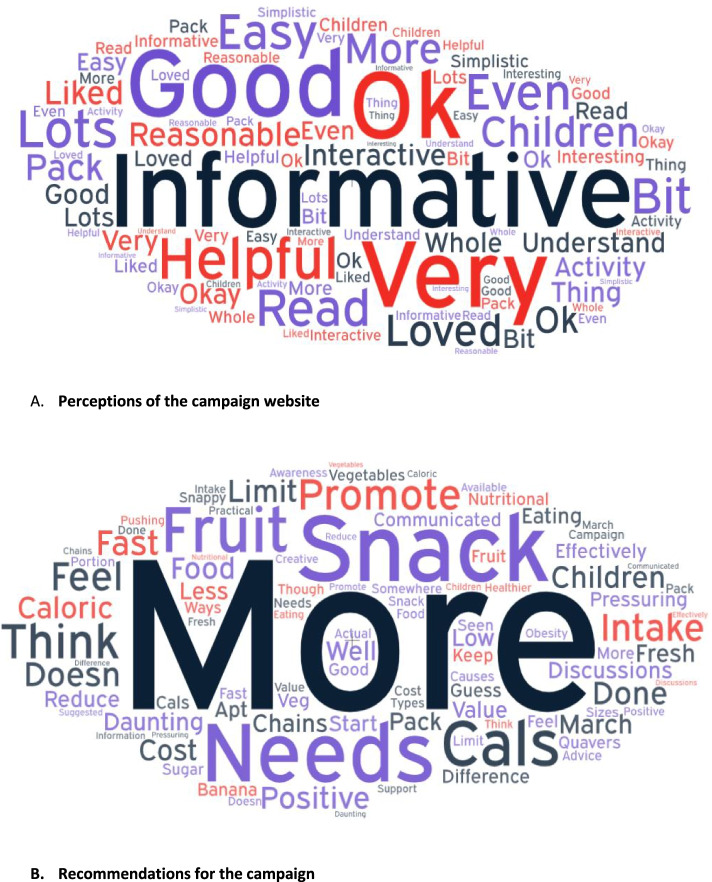
Word clouds highlighting parent perceptions of the C4L 100 cal snack campaign

When the respondents were asked. ‘please tell us what you thought about the C4L 100 cal snack campaign overall? 132 respondents (who had seen the campaign) provided a written response. Figure 2 highlights these perceptions. The following themes emerged from their feedback; positive views on the campaign (overall good acceptance and positive impact); negative views on the campaign (poor acceptance of campaign messages); recommendations for improvements to the campaign.

Just over half of these respondents used positive language to describe the campaign; describing it as good, very good, effective, useful, helpful or informative/interesting (*n* = 77, 58.3%). For example: *“It was a brilliant help with snack ideas to give my children. It gave me a different variety of snack ideas which were very healthy for them”;* “*This campaign is a very good idea. It can help parents to care more about what their children eat.”*

A few respondents believed it was eye-catching, memorable and easy to remember. For example: *“The brightly coloured leaflet and posters draws people’s attention to it, so people are more willing to learn about the campaign and read the information”*. Others stated that they would like to have seen more examples of actual recommended snacks.

Furthermore, a few respondents reported a positive impact of the campaign, with improved awareness of healthier nutrition and making healthier snack choices. For example: *“made me really think about what I can give as snacks and trying new things”.* Some also reported that their children were receptive to the campaign. For instance: *“It was appealing to my daughter as she was able to make healthy choices in the supermarket”.* Conversely, some respondents indicated their disagreement with the campaign messages focussing predominantly on calorific content of snack foods, as well as perceiving poor suitability of snack examples. This is illustrated by the following examples:

"It is short sighted and unhelpful to suggest that low calorie snacks are the best snacks, or that processed snack foods full of sweeteners are a good alternative to proper full foods"

"I do not agree with the campaign. I give my child nutritious snacks. The calorific value is not important. I do not want to teach my children to count calories, but to eat intuitively"

Some respondents also suggested recommendations for improvements to the campaign, for example, increased promotion and more information on healthy snack choices, or for an alternative focus. This is illustrated with the following quotations from respondents:

“More information needs to be available on snack types and portion sizes”

"We should be promoting only fruit and veg as snacks"

"A low sugar campaign would be more apt as this is what causes obesity"

### About the 100 cal snack information website

The survey asked, ‘did the campaign encourage you to search for 100 calorie snack information on the website?’, to which 78.3% (*n* = 141,) reported that it did not. The survey also asked ‘what did you think about the 100 calorie snack information on the website?’ Twenty-seven people commented, with a majority describing the website as good, informative or just okay (as highlighted in Fig. 2). Moreover, a few parents indicated that the information on the website was helpful:

*“Good ideas for healthy snacks”.*

*“Really like recipe ideas for lunchboxes”.*

Respondents were asked for their agreement with a series of statements about the information available on the C4L 100 cal snack website (only 39 respondents reported actually seeing the website). Of the respondents who completed these statements three quarters ‘agreed’ or ‘strongly agreed’ that the website informed them about 100 cal snacks (*n* = 30, 77%). The majority agreed that the examples of snacks were useful (*n* = 34, 87%), but less than half agreed that the examples of snacks were easy to make at home (*n* = 18, 47%). Just over half agreed that the snacks were affordable (*n* = 19, 51%) and that their children liked the examples of snacks (*n* = 19, 51%). Most ‘agreed’ or ‘strongly agreed’ that the 100 cal snack information was easy to understand (*n* = 34, 85%) and nearly three quarters ‘agreed’ or ‘strongly agreed’ that it helped them to understand what a healthy snack looked like (*n* = 28, 74%). Around two-thirds reported agreeing or strongly agreeing that the information helped them to find calorie information on packaging (*n* = 25, 64%).

### Perceived impact of the 100 cal snack campaign on snacking behaviours

There was no clear consensus of a perceived positive impact on healthier snack purchasing nor preparing more 100 cal snacks at home. However, some respondents reported making positive changes:

*“It made a huge difference to my family’s eating habits”.*

Of the 40 respondents to complete the question, a similar number of respondents ‘agreed’ (*n* = 10, 25%), ‘disagreed’ (*n* = 11, 28%) or ‘neither agreed nor disagreed’ (*n* = 13, 33%) that they now buy more 100 cal snacks when shopping. Of the 39 respondents who responded to the question about whether they now prepare more 100 cal snacks at home, a similar number of respondents ‘agreed’ (*n* = 11, 28%), ‘disagreed’ (*n* = 9, 23%) or ‘neither agreed nor disagreed’ (*n* = 14, 36%). Respondents did, however, report that they looked at the nutritional information on packaging more frequently due to the campaign, for around a half (*n* = 24, 52%) ‘agreed’ or ‘strongly agreed’ that they now look for calorie information on packaging and just under two thirds (*n* = 24, 63%) ‘agreed’ or ‘strongly agreed’ that they now look at traffic labelling on packaging.

Respondents were then asked about their child’s frequency of snack consumption. A greater number of respondents were able to answer the questions that were not directly related to the campaign. When asked how many times in 1 day respondents give their child a snack (not including fruit and vegetables), of the 318 respondents to the question, a mean of 1.7 (SD, 1.0) times per day was given. When asked how many times in 1 day respondents give their child fruit and/or vegetables as a snack, of the 319 respondents to the question, a higher mean of 2.0 (SD, 1.2) times per day was given. The following questions related to changes in snack consumption since the campaign specifically. A much lower number of respondents completed these questions (*N* = 65), as many had not seen the campaign. Nearly two thirds reported no change in number of times their child consumed a snack per day (not including fruit and/or vegetables) since seeing the campaign (*n* = 41, 63%), with only 11% (*n* = 7) reporting that it had decreased. Most reported that the number of times their child consumed fruit and/or vegetables as a snack per day since seeing the campaign, had stayed the same (62%, *n* = 40), with only 15% reporting an increase in fruit and vegetable consumption (*n* = 10).

### Improvements and recommendations to the campaign

When asked about their perceptions around improvements to the 100 cal snack information (for example type of information, how it looks, where you find it), 89 respondents provided a written response. The following themes emerged from the feedback: promotion of the campaign, recommended snack examples and nutritional labelling.

For example, around half of the comments related to better advertising and publicity around the campaign. Some examples were provided and included mainly delivering through educational settings (school, nurseries), social media, television/radio and at supermarkets. Figure 2 highlights these perceptions.

Around a quarter of respondents suggested improvements to snack products. Some comments related to improved healthiness of snack food ideas, with a handful of respondents disliking artificial sweeteners in low sugar and low fat examples, with a preference for real whole foods. For example:

“Sugar free items that are sweet are full of other chemicals which I prefer not to give my child. It would be better to suggest snacks that are made from non 'snack foods' already in the house, like a small peanut butter sandwich on wholemeal bread, which I suppose might be more than 100 calories depending on how its made, so advice on this type of snack would be useful"

Several wanted more specific ideas for healthier snacks, with examples being more visible in the campaign. A few comments related to improved labelling of products, to make it clearer which products meet the 100 cal guidelines, for example:

"It might be helpful…if there was something indicating snacks that are under 100 calories on the shelves. It would possibly lead to people making more informed choices for snacks and lunchbox fillers"

Several respondents disliked the target message of calories, occasionally perceiving calorie counting to be ill-advised for children, preferring an alternative focus on overall healthiness of diet, for example:

“Don't focus on calories - it's not health …would it not be better to have categories…we have allergy children (dairy and egg so focus on healthy snacks for calcium, iron, iodine, zinc, etc). We need to step away from quantifying the item and look at the quality"

Some thought focussing on sugar content or portion sizes could be more suitable. A few comments also related to making the campaign more appealing to children, through use of apps, games, posters with tick boxes for when a snack is eaten, for example:

“Top trump cards for children to play with categories such as 'sugar content, calories, dental health' values”

### Supporting parents to provide healthier snacks for their children

Respondents were asked how they would like to be supported to provide healthier snacks for their children. One hundred and twenty four respondents commented. The following themes emerged from the discussion: improved access to, availability of and display of healthier snack items; clearer nutritional labelling; creating more opportunities for children to eat more healthily and more information and guidance around healthy eating.

Around a quarter of comments related to strategies for improved access to healthier snacks in supermarkets/shops. These included more availability and choice of healthier snack products (low sugar, low salt, low fat) and improved display of healthier products (less visibility of high sugar high fat options), for example:

“Create aisle ends - dedicated areas for healthier snacks in supermarkets” “Supermarket to make a specially selected snack items corner with free tasting samples”

Respondents desired better promotion of and more information on low sugar, low fat, low salt options in supermarkets and shops, as well as increased availability of healthier options at other venues such as cafes, leisure centres, vending machines, cinemas, theme parks etc. Others desired increased availability of cheaper, low sugar and low-fat snack options and fruits and vegetables and money-off vouchers for healthy foods made available. For example:

"I can easily find whole isles of chocolate and crisps, but healthy crackers for example are hard to find and expensive"

"Should be more fresh fruit and healthy snacks on offer at cinemas, theme parks, child friendly outings"

Some wanted clearer nutritional information labelling on the packaging, particularly calories and sugar, portion sizes and allergen information. Others discussed the need for tighter restrictions on marketing of high sugar high fat items to children, with television characters used for promoting healthier snack items, for example:

“Child friendly packaging and more obvious sugar warning signs”

“Ban food manufacturers from promotions with toy/tv/film characters/companies unless it’s a healthy snack”

Some respondents perceived that schools or nurseries would be useful environments for targeting children, by improving packed lunches (for example with prizes for best lunchbox), providing healthier meals, and restricting sales and provision of high sugar, high fat items on site and creating more opportunities for children to try new healthier foods. For example:

"Schools should take on board the information as my child is given high calorie snacks in the form of cupcakes/sweets provided as a reward for good behaviour or volunteering"

“Schools to follow their healthy campaigns through by looking at the sugar/fat content of their school dinners better. Nurseries to have better training/guidelines on healthy options for children”

Around a quarter of respondents wanted improved guidance and information on healthier snack provision for their children. Many of which related to more information on healthy snack choices (low sugar mainly), for example, healthy carbohydrate based snacks, suitable easy ideas and recipes for children, such as sugar free treat recipes, and also ideas that can be prepared and stored in advance. Providing information (for example a list of healthy snacks ideas) by emails, leaflets, Apps, or on a snack chart was recommended. A few wanted reminders around eating healthily as well. Several wanted ideas on how to encourage fussy eaters to eat more healthily with filling low sugar tasty options. For example:

"Sometimes it is hard as a parent to encourage your child to eat healthier - my youngest would choose a sugary treat over something healthier although does try"

“Hints and tips on how to encourage children to try healthy foods”

"A campaign that shows me the products so it is quick and easy to identify when shopping or ordering online”

The survey then asked how they would like information about healthy snacking to be provided. Ninety three respondents provided a written response. Themes related to improved delivery and promotion of information and strategies for better nutritional labelling. Many comments related to preferred methods for delivery of information, with ‘through school’ being the most popular. Other suggestions included TV advertising (or radio for older generations), emails and websites, social media, applications on mobile phones, in supermarkets or stores and leaflets. Some also commented on the need for clearer nutritional labelling on product packaging, regarding the healthiness of products, for example, clear labelling at the front of the package showing important nutritional information that can be easily and quickly interpreted, e.g. through traffic light labelling.

"Make it statutory for price labelling as well as packaging to be given the same green light logo to make it stand out more"*.*

"Traffic lights easy to view at a glance to make quick decision. Not much reading done by colour"

There was also a suggestion for traffic light labelling to extend to take-away packaging and for artificial sweeteners to be clearly labelled on the packaging. A few expressed the difficulty with knowing what healthy snacks to give to children and thus wanted ideas for healthy snacks, easy to follow and easily accessible recipes (e.g via an App), that children can also follow as well.

### Acceptable initiatives to support parents to choose healthier snacks for their children

The survey presented a list of strategies for providing more 100 cal snacks for children and respondents were asked to select which ones were most acceptable to them (see the full survey in the supplementary materials). Respondents were able to select as many strategies as they wished. Of the 550 statements selected, the most popular strategy was a sticker or logo that states the following product meets the 100 cal guidelines (*n* = 192, 34.9%), followed by more products in 100-cal portions (*n* = 164, 29.8%) and easier labelling on which products are 100 cal (*n* = 161, 29.2%). A few comments related to focussing less on calorie content of pre-packaged snacks, but rather providing ideas for healthier snacks made from ‘real whole foods (as opposed to processed items), appropriate portion sizes, and other alternative ideas to just fruit and vegetables for snacks. For example:

“I have seen snacks advertising that they have less than 100 calories but they aren’t necessarily healthy e.g. crisps or iced gems… But I wish there were more easy, low sugar, healthy options”*.*

“Ideas above seem to be focussed on pre-packaged / processed foods which I would prefer to avoid, so more ideas about home-prepared snacks or portion sizes eg of crackers, breadsticks, hummus etc*.*

Respondents could also select from lists of initiatives to help parents provide healthier choices for their children, which would be most acceptable to them (they could select as many options as they wished, 822 statements were selected). The most popular strategies were ‘healthy snack ideas that are easy to prepare (*n*=241, 29.3%) and ‘making healthier products cheaper than less healthy ones’ (*n*=231, 28.1%); followed by ‘providing fruit and vegetables that are more affordable’ (*n* = 190, 23.1%) and ‘all packaged products using traffic light labelling’ (*n* = 146, 17.8%). Of a list of further strategies presented (300 statements selected), the most popular strategy was ‘replacing unhealthy products near the checkouts with healthier ones’ (*n* = 87, 29.0%). Similar lower proportions of respondents preferred the following strategies: ‘changing ingredients in food gradually so people don’t notice a change in taste’ (*n* = 53, 17.7%), ‘changing ingredients in food to reduce the calories or amount of sugar, though this may change the taste of the product’ (*n* = 52, 17.3%), ‘reducing the size of the unhealthy products and keeping the same price’ (*n* = 48, 16.0%) and ‘reducing the size of unhealthy products and reducing the price’ (*n* = 47,15.7%).

Other recommendations (*n* = 27) included the following: cheaper, healthier, age-appropriate options for children; greater availability of healthier snacks; snacks that stay in date for longer; more affordable fruit and vegetables in good condition; make foods more natural and less sweet; sugar free snacks not full of additives or sweeteners; make healthier products taste good for children, including more “kid friendly” vegetable foods; “grab and go” ideas that do not need preparation; suggestions for filling meals to prevent snacking; fruit and vegetable snacks beside tills; Change4Life tuck shop in schools; more recyclable packaging. Ideas for other more top-down approaches included: limit snack calorie sizes by legislation; regulate advertising of HFSS foods aimed at children and advertising aimed at grandparents about healthy eating/snacking.

## Discussion

To the author’s knowledge, this is the first study to explore parents’ perceptions of the C4L 100 cal snack campaign and to explore its perceived impact on snack intake in families. Previous evaluations of social marketing interventions targeted at adults [34, 35] and children [36], suggest that they are a good approach to share information. Our findings indicate a moderate awareness of the C4L 100 cal snack campaign, (just over half of respondents), with many of those stating that they had seen the C4L leaflet or a television advert at least once. There was a greater awareness of the campaign in our sample than indicated in an earlier online panel survey, commissioned by PHE in January 2018 [29], where only a third of their sample reported being aware of the phrase ‘look for 100 calorie snacks, two a day max’ (*n* = 47 respondents aged 16–75 years). Awareness of the broader C4L campaign has been reported to be greater in a previous cluster-based randomised controlled trial examining its impact on parents’ attitudes and behaviours about their children’s eating and activity (75% at baseline) [27]. The lower level of awareness of the 100-cal snack campaign overall in our sample may be attributed to the small timeframe for mass media campaign promotion (only 2 months). Most respondents agreed that the campaign caught their attention, had informed them about 100 cal snacks and that they thought it was memorable. Such findings are positive since well-performing campaigns and adverts are attention-grabbing and stand out against a crowd of other information [37]. Many respondents agreed that the campaign made them think about limiting high sugar and high fat snack foods for their child, and just under a half agreed that it made them think about dental decay in their child. Such findings are in line with other studies of social marketing campaigns that have found positive effects on attitudes towards target behaviours [38], including an evaluation of the C4L smart-swaps campaign [39]. By encouraging parents and carers to think about the foods they are giving their children, the C4L100 cal snack campaign may also help to strengthen intentions to alter behaviour and increase the likelihood of achieving new, healthier snack behaviours [40].

Although some respondents stated that the C4L website had improved their awareness of healthier snacks, most respondents indicated that they were not aware of the campaign website and were not aware of the 100 cal information available on the site. As levels of behaviour change have been correlated with campaign exposure [36], this finding may suggest that more emphasis on campaign dissemination and/or promotion is needed. The increased promotion was also supported by respondents in their comments about improving the campaign. Furthermore, the 40 respondents that reported their perceptions of the 100 cal website information, mainly agreed that the website information was easy to understand and it helped them to understand what a healthy snack looked like, and the examples of snacks provided were useful, indicating that increased publicity and signposting to the website information and specifically the snack examples, would be beneficial. As only around half thought that the snacks were easy to prepare at home or affordable, perhaps some modifications to the snack examples need to be made to provide a larger range of affordable and easily prepared examples.

In terms of the perceived impact of the campaign information on family snack habits, there was no clear consensus of a perceived positive impact on healthier snack purchasing nor preparing more 100 cal snacks at home. This supports previous research indicating that the C4L mass media campaign had little impact on attitudes or behaviours towards healthy eating [27]. Whilst some respondents stated that they had increased the frequency with which fruit and/or vegetables were given to children as a snack since seeing the campaign, many reported no change. Whilst the characteristics of the respondents in each group were not explored, previous research indicates that short term behavioural changes to health campaigns occur mainly in highly motivated individuals [41]. Respondents did however report looking at the nutritional information on packaging more frequently due to the campaign, which could result in positive changes to intake in the longer term.

The qualitative findings from the survey indicate that most parents and carers were positive about the campaign. However, some indicated concern about the focus on calories over health, the lack of consideration of the variable nutrition needs across children, and the poor suitability of snack examples. Other comments relating to improvements to the campaign included more specific ideas for healthier snacks and consideration of appropriate portion sizes for children. Some respondents stated that additional strategies should be considered such as improved access to and marketing of healthier snacks in supermarkets and shops and clearer nutritional labelling. Research has shown in-store strategies such as modifying product availability, placement and promotion are effective in reducing sales of unhealthy discretionary foods [42–44].

Findings from this study point to several recommendations to policymakers, food manufacturers and intervention developers. The recommendations are based on the aspects of the C4L campaign that parents found most useful and also those aspects that they found were lacking. Some of the recommendations provided were explicitly stated by parents, others were developed by the research team based on the analysis of the overall survey data set and through reflection of this data with previous research including that by Economos et al. [45] which assessed effective social marketing campaigns for healthy eating. The recommendations from the current study are presented below, alongside the key areas for action identified by the previous research.

1. Recommendation 1: future campaigns should seek to increase availability of more affordable low sugar, low fat, low salt options, including more fruit and vegetables in food outlets including cafes, restaurants, and recreational venues. Money-off vouchers for healthier options and providing a larger range of more healthful products in 100 cal portions could be helpful.
2. Recommendation 2: parents should be supported to make healthier choices for their children by improving visibility and promotion of healthier snacks with improved displays. For example, by placing healthier snacks and fruits and vegetables at the checkouts, at the end of aisles, in designated sections, and always clearly labelled as healthier options. Less visibility of high sugar, high fat, high salt options should also be promoted.
3. Recommendation 3: clear nutritional labelling on the front of the packaging, (including calories and portion size) needs to be included. Moreover, easy and quickly interpretable indicators of healthiness (sugar, fat, salt) of the product should be present, for example through universal traffic light labelling. Traffic light labelling should be extended to fast food/take-away outlets. Additionally, labelling on which products meet the 100 cal snack guidelines would be beneficial.
4. Recommendation 4: 100 cal snack campaign could be made more appealing to children through the use of child-friendly materials, recommended examples include apps, games, posters and healthy snack charts. We also recommend that healthier (low sugar, low fat, low salt) foods and drinks targeting children, are marketed with the use of popular television characters or similar.
5. Recommendation 5: all messages should be pretested to ensure they are framed and understood correctly. Materials to be tested could include healthy recipes and pre-prepared snack options. The recipes and ideas should be easily accessible, child-friendly, quick to prepare, and low in sugar and primarily based on whole foods rather than processed foods.
6. Recommendation 6: information on healthy snacking should be well distributed and published, made available to parents through a range of media, delivered through school/educational settings, online (emails, Apps, websites), TV/radio advertising, social media, supermarkets (posters, leaflets) and leaflets.

These recommendations reflect many of the key points highlighted by Economos et al. (2001) including the importance of economic issues and making changes seem convenient, non-sacrificial and low cost; that industry should be involved in campaigns and should be pushed to set better food standards; that key legislators who are sensitive to the issue should be involved and all partners should benefit (e.g. government, industry, consumers); and that the message of the campaign needs to be right, with mass media engaged with.

### Strengths and limitations

Whilst the study provides important and unique insights into the perceptions, awareness, and potential impact of the C4L 100 cal snack campaign, which are of interest to policymakers and researchers, it is not without limitations. First, whilst efforts were made to recruit a diverse population of parents and carers from across the UK, the sample was predominately from the north of the UK, most were white females and of a high educational level. The proportion of respondents from the 20% most deprived areas was however similar to the national average. As such the findings may not be representative of the perceptions of the whole UK population, and lack representation from fathers and communities of lower socioeconomic status and varying ethnic backgrounds. Establishing key contacts working in diverse communities to support the promotion of the survey (for example a council public health team) could help overcome this in future research. Respondents were asked to self-report snack and fruit and vegetable consumption which can be compromised by self-report bias [46]. Also, as the questions were optional, some respondents missed several of the questions. Whilst this may have helped to improve overall response and survey completion rates, it is possible that in skipping the questions, some bias may have been introduced. Furthermore, the views around acceptability and perceptions of the campaign were provided from a small subset of the population. Whilst our findings indicate that awareness of the campaign was only moderate overall, which is an important finding, this resulted in a small number of respondents for some questions, meaning that the findings may lack generalisability to the whole UK population. Furthermore, conducting qualitative analysis is subject to potential bias. To minimise this, two researchers conducted the qualitative analysis and organised the data into themes. Moreover, a summary of all of the qualitative findings are presented, and all the themes (major and minor) discussed, not just a selection of the results, with quotes used to illustrate key findings to improve validity. Moreover, as behaviour change is typically a slow process, it is important to assess the impact of the C4L 100 cal snack campaign, and related initiatives over a longer follow-up period.

## Conclusion

This study indicates that although around half of the sample had some awareness of the C4L 100 cal snack campaign, many respondents indicated that the campaign materials had little impact on attitudes or behaviours related to their children’s snacking. Some suggestions for improvements to extensions of the C4L 100 cal snack campaign were provided to create long term behaviour change. For instance, some families welcomed additional development of children’s nutrition interventions based on the C4L 100 cal snack campaign including clearer advertising towards health snacks and labelling and promotion of healthy snack ideas for children. Such insights could help to increase the long term impact of this campaign and improve the success of future children’s nutrition interventions. Future social marketing campaigns could be improved through the use of more formal pilot testing to assess the understanding and acceptance of the campaign amongst the target audience. Further research is needed to explore the perceptions of the C4L 100 calorie snack campaign amongst a broader spread of the population, including children.

## Supplementary Information

**Additional file 1.**

## Data Availability

The datasets used and/or analysed during the current study are available from the corresponding author on reasonable request.

## Abbreviations

UK: United Kingdom
NCMP: National Child Measurement Programme
NDNS: National Diet and Nutrition Survey
C4L: Change4Life
PHE: Public Health England
NCDs: Non-communicable diseases
IMD: Index of Multiple Deprivation
App: Application on a mobile device
