# Supplementary material for: Parents’ awareness and perceptions of the Change4Life 100 cal snack campaign, and perceived impact on snack consumption by children under 11 years
Source: BMC Public Health. 2022 May 19;22:1012. doi: 10.1186/s12889-022-12789-7 (PMC9118772; doi:10.1186/s12889-022-12789-7)
Supplement: Supplementary file 1 — Additional file 1. [file 12889_2022_12789_MOESM1_ESM.docx]

| Q4_1 | Q4_2 | Q4_3 | Q4_4 | Q5 | Q6 | Q7 | Q7_3_TEXT | Q9_1 | Q9_2 | Q9_3 | Q9_4 | Q10_1 | Q10_2 |
| --- | --- | --- | --- | --- | --- | --- | --- | --- | --- | --- | --- | --- | --- |
| How often does everyone who lives in your house eat these meals together? (please select one option per eating occasion) - Breakfast | How often does everyone who lives in your house eat these meals together? (please select one option per eating occasion) - Lunch | How often does everyone who lives in your house eat these meals together? (please select one option per eating occasion) - Evening meal | How often does everyone who lives in your house eat these meals together? (please select one option per eating occasion) - Snacks | When you eat your evening meal together, approximately what time do you usually eat? | When you eat your evening meal together, how long does the meal usually take? | Is your youngest child currently at school, pre-school/ nursery (under 5 years old)? - Selected Choice | Is your youngest child currently at school, pre-school/ nursery (under 5 years old)? - Other, please state - Text | How often do you eat meals with your youngest child? - Breakfast | How often do you eat meals with your youngest child? - Lunch | How often do you eat meals with your youngest child? - Evening meal | How often do you eat meals with your youngest child? - Snacks | During the week, if your child eats at home, where do they most frequently eat the following meals? (please tick one option for each eating occasion) - Breakfast | During the week, if your child eats at home, where do they most frequently eat the following meals? (please tick one option for each eating occasion) - Lunch |

| Q10_2 | Q10_3 | Q10_4 | Q67 | Q66_1 | Q66_2 | Q66_3 | Q66_4 | Q62 | Q13_1 |
| --- | --- | --- | --- | --- | --- | --- | --- | --- | --- |
| During the week, if your child eats at home, where do they most frequently eat the following meals? (please tick one option for each eating occasion) - Lunch | During the week, if your child eats at home, where do they most frequently eat the following meals? (please tick one option for each eating occasion) - Evening meal | During the week, if your child eats at home, where do they most frequently eat the following meals? (please tick one option for each eating occasion) - Snacks | If you selected other, where does your child eat during the week? | At the weekend, if your child eats at home, where do they most frequently eat the following meals? (please tick one option for each eating occasion) - Breakfast | At the weekend, if your child eats at home, where do they most frequently eat the following meals? (please tick one option for each eating occasion) - Lunch | At the weekend, if your child eats at home, where do they most frequently eat the following meals? (please tick one option for each eating occasion) - Evening meal | At the weekend, if your child eats at home, where do they most frequently eat the following meals? (please tick one option for each eating occasion) - Snacks | If you selected other, where does your child eat at the weekend? | During the week, if your child eats away from the home, where do they eat most frequently the following meals? (please tick one option for each eating occasion) - Breakfast |

| Q13_2 | Q13_3 | Q13_4 | Q63 | Q68_1 | Q68_2 | Q68_3 | Q68_4 | Q69 | Q15_1 |
| --- | --- | --- | --- | --- | --- | --- | --- | --- | --- |
| During the week, if your child eats away from the home, where do they eat most frequently the following meals? (please tick one option for each eating occasion) - Lunch | During the week, if your child eats away from the home, where do they eat most frequently the following meals? (please tick one option for each eating occasion) - Evening meal | During the week, if your child eats away from the home, where do they eat most frequently the following meals? (please tick one option for each eating occasion) - Snacks | If you selected other, where does your child eat when away from the home during the week? | At the weekend, if your child eats away from the home, where do they eat most frequently the following meals? (please tick one option for each eating occasion) - Breakfast | At the weekend, if your child eats away from the home, where do they eat most frequently the following meals? (please tick one option for each eating occasion) - Lunch | At the weekend, if your child eats away from the home, where do they eat most frequently the following meals? (please tick one option for each eating occasion) - Evening meal | At the weekend, if your child eats away from the home, where do they eat most frequently the following meals? (please tick one option for each eating occasion) - Snacks | If you selected other, where does your child eat when away from the home at the weekend? | How often does your youngest child watch a screen while eating? (please tick one option for each eating occasion) - Breakfast |

| Q15_2 | Q15_3 | Q15_4 | Q16_1 | Q16_2 | Q16_3 | Q16_4 | Q17 | Q18_1 | Q18_2 |
| --- | --- | --- | --- | --- | --- | --- | --- | --- | --- |
| How often does your youngest child watch a screen while eating? (please tick one option for each eating occasion) - Lunch | How often does your youngest child watch a screen while eating? (please tick one option for each eating occasion) - Evening meal | How often does your youngest child watch a screen while eating? (please tick one option for each eating occasion) - Snacks | How often does your youngest child eat approximately the same types of food as you for a snack? (Please tick one option) - Breakfast | How often does your youngest child eat approximately the same types of food as you for a snack? (Please tick one option) - Lunch | How often does your youngest child eat approximately the same types of food as you for a snack? (Please tick one option) - Evening meal | How often does your youngest child eat approximately the same types of food as you for a snack? (Please tick one option) - Snacks | How important is it to you that you eat meals as a family? (Please tick one option) | How many portions of fruit and vegetables does your youngest child have at each meal? (A portion or serving is a handful of fruit or vegetables) - Breakfast | How many portions of fruit and vegetables does your youngest child have at each meal? (A portion or serving is a handful of fruit or vegetables) - Lunch |

| Q18_3 | Q18_4 | Q21 | Q22 | Q22_8_TEXT | Q23 | Q24 | Q24_9_TEXT | Q25 |
| --- | --- | --- | --- | --- | --- | --- | --- | --- |
| How many portions of fruit and vegetables does your youngest child have at each meal? (A portion or serving is a handful of fruit or vegetables) - Evening meal | How many portions of fruit and vegetables does your youngest child have at each meal? (A portion or serving is a handful of fruit or vegetables) - Snacks | Have you come across the campaign “look for 100 calorie snacks, two a day max’? | Where have you seen or heard the phrase ‘look for 100 calorie snacks, two a day max’? (Select as many responses as appropriate) - Selected Choice | Where have you seen or heard the phrase ‘look for 100 calorie snacks, two a day max’? (Select as many responses as appropriate) - Other, please state - Text | Have you seen or received a leaflet about the 100 calorie snack campaign? | If yes, where did you see or receive this leaflet? - Selected Choice | If yes, where did you see or receive this leaflet? - Other, please state - Text | Overall, how many times in total did you see “look for 100 calorie snacks, two a day max” advertised? |

| Q27_1 | Q27_2 | Q27_3 | Q27_4 | Q27_5 | Q27_6 | Q27_7 | Q27_8 | Q28 |
| --- | --- | --- | --- | --- | --- | --- | --- | --- |
| Please state your agreement with the following statements - The campaign caught my attention | Please state your agreement with the following statements - The campaign informed me about 100 calorie snacks | Please state your agreement with the following statements - The campaign was appealing (looked good) | Please state your agreement with the following statements - The campaign was convincing | Please state your agreement with the following statements - The campaign was memorable | Please state your agreement with the following statements - It made me think about limiting my child’s sugar intake | Please state your agreement with the following statements - It made me think about limiting high sugar and high fat snack foods for my child | Please state your agreement with the following statements - It made me think about dental decay in my child | Did the campaign encourage you to search for 100 calorie snack information on the website? |

| Q29 | Q30 | Q31 | Q32 | Q33_1 | Q33_2 | Q33_3 | Q33_4 | Q33_5 | Q33_6 | Q43 |
| --- | --- | --- | --- | --- | --- | --- | --- | --- | --- | --- |
| Did the campaign encourage you to sign up for money-off vouchers for healthier snack options? | Please tell us what you thought about the Change4Life 100 calorie snack campaign overall? | Have you seen the 100 calorie snack information on the Change4Life website? | What did you think about the 100 calorie snack information on the website? | Please state your agreement with the following statements - The website informed me about 100 calorie snacks | Please state your agreement with the following statements - The examples of snacks were useful | Please state your agreement with the following statements - The examples of snacks were easy to find when shopping | Please state your agreement with the following statements - The examples of snacks were easy to make at home | Please state your agreement with the following statements - The examples of snacks were affordable | Please state your agreement with the following statements - My child liked the examples of snacks | Has the number of times you give your child a snack (NOT including fruits and vegetables) changed since you have seen the campaign? |

| Q45 | Q40_1 | Q40_2 | Q40_3 | Q42_1 | Q42_2 | Q42_3 | Q42_4 | Q34 | Q35 |
| --- | --- | --- | --- | --- | --- | --- | --- | --- | --- |
| Has the number of times you give your child a fruits and vegetables as a snack changed since you have seen the campaign? | Please state your agreement with the following statements - The 100 calorie snack information was easy to understand | Please state your agreement with the following statements - It helped me to understand what a healthy snack looks like | Please state your agreement with the following statements - It helped me to understand where to find calorie information on food packaging | Please state your agreement with the following statements - I now buy more 100 calorie snacks when shopping | Please state your agreement with the following statements - I now prepare more 100 calorie snacks at home | Please state your agreement with the following statements - I now look for calorie information on packaging | Please state your agreement with the following statements - I now look at traffic light labelling on packaging | The website encouraged me to sign up to Change4Life | The website encouraged me to download the Food Scanner App |

| Q36 | Q37 | Q38 | Q41_1 | Q44_1 | Q46_1 | Q46_2 | Q46_3 | Q46_4 |
| --- | --- | --- | --- | --- | --- | --- | --- | --- |
| Have you used the Food Scanner App when shopping? | How often have you used the Food Scanner App when shopping? | What do you think about the Food Scanner App? | How many times in one day do you give your child a snack (NOT including fruit and vegetables)? - Select the number of snacks given per day | How many times in one day do you give your child fruit or vegetables as a snack? - Select the number of times fruit and veg are given as snacks per day | Out of the following snacks recommended on the Change4Life 100 calorie snack website, which have you given your child before seeing the campaign or since seeing the campaign? Please select one response per question from the drop down list - Malt loaf slice | Out of the following snacks recommended on the Change4Life 100 calorie snack website, which have you given your child before seeing the campaign or since seeing the campaign? Please select one response per question from the drop down list - Fromage frais (lower fat and lower sugar) | Out of the following snacks recommended on the Change4Life 100 calorie snack website, which have you given your child before seeing the campaign or since seeing the campaign? Please select one response per question from the drop down list - Fresh or tinned fruit salad | Out of the following snacks recommended on the Change4Life 100 calorie snack website, which have you given your child before seeing the campaign or since seeing the campaign? Please select one response per question from the drop down list - Plain rice cakes or crackers with lower-fat cheese |

| Q46_5 | Q46_6 | Q46_7 | Q46_8 | Q46_9 | Q47 | Q48 | Q49 | Q49_4_TEXT |
| --- | --- | --- | --- | --- | --- | --- | --- | --- |
| Out of the following snacks recommended on the Change4Life 100 calorie snack website, which have you given your child before seeing the campaign or since seeing the campaign? Please select one response per question from the drop down list - Sugar-free jelly | Out of the following snacks recommended on the Change4Life 100 calorie snack website, which have you given your child before seeing the campaign or since seeing the campaign? Please select one response per question from the drop down list - One scotch pancake | Out of the following snacks recommended on the Change4Life 100 calorie snack website, which have you given your child before seeing the campaign or since seeing the campaign? Please select one response per question from the drop down list - Fresh, frozen or tinned vegetables | Out of the following snacks recommended on the Change4Life 100 calorie snack website, which have you given your child before seeing the campaign or since seeing the campaign? Please select one response per question from the drop down list - Boiled eggs | Out of the following snacks recommended on the Change4Life 100 calorie snack website, which have you given your child before seeing the campaign or since seeing the campaign? Please select one response per question from the drop down list - Popcorn | Can you recommend any improvements to the 100 calorie snack information? e.g. type of information, where you can find the information, the way it looks etc | How would you like to be supported to provide healthier snacks for your children? | Which of the following would help you provide more 100 calorie snacks for your children? (Select as many as you wish) - Selected Choice | Which of the following would help you provide more 100 calorie snacks for your children? (Select as many as you wish) - Other, please state - Text |

| Q50 | Q50_10_TEXT | Q64 | Q64_6_TEXT | Q51 | Q52_1 | Q53_1 | Q53_2 | Q53_3 | Q53_4 | Q54 |
| --- | --- | --- | --- | --- | --- | --- | --- | --- | --- | --- |
| Which of the following initiatives would help parents to provide healthier snacks for their children? - Selected Choice | Which of the following initiatives would help parents to provide healthier snacks for their children? - Other, please state - Text | Which of the following initiatives would be acceptable to you? - Selected Choice | Which of the following initiatives would be acceptable to you? - Other, please specify - Text | Please tell us how would you like information about healthy snacking to be provided | How old is your youngest child? Please select their age - What is their age in years? | How old are your other children? - Child 2 | How old are your other children? - Child 3 | How old are your other children? - Child 4 | How old are your other children? - Child 5 | What role do you play in the family? - Selected Choice |

| Q54_5_TEXT | Q55 | Q55_3_TEXT | Q56_1 | Q57 | Q57_6_TEXT | Q58 | Q58_8_TEXT | Q59 | Q59_15_TEXT |
| --- | --- | --- | --- | --- | --- | --- | --- | --- | --- |
| What role do you play in the family? - Other, please state - Text | How would you describe your gender? - Selected Choice | How would you describe your gender? - Other, please state - Text | What is your age in years? - Select your age | What is your marital status? - Selected Choice | What is your marital status? - Other, please state - Text | What is your current employment status? - Selected Choice | What is your current employment status? - Other, please state - Text | What is your highest qualification? - Selected Choice | What is your highest qualification? - Other, please state - Text |

| Q60 | Q61 | Q61_2_TEXT | Q61_3_TEXT | Q72 | Q72_18_TEXT | Q70 | Q75 | Q65 |
| --- | --- | --- | --- | --- | --- | --- | --- | --- |
| Which best describes the total annual income of your household (before tax and deductions, but including benefits/allowances)? | Which country were you born in? - Selected Choice | Which country were you born in? - Other country, please state - Text | Which country were you born in? - When did you start living in the UK? - Text | How would you describe your ethnic background? - Selected Choice | How would you describe your ethnic background? - Any other ethnic group, please state - Text | What is your postcode? (Please input as follows: LS16 7RS) | We are currently looking for parents with children aged up to 11 years old, to take part in a 30-minute focus group in the Leeds area. If you would like further information about this, please write your email address. We will not share your email address with third parties. | If you would like to be included into the prize draw, please state your email address. We will not share your email address with third parties. |
